# How significant to plant N nutrition is the direct consumption of soil microbes by roots?

**DOI:** 10.1111/nph.12320

**Published:** 2013-05-30

**Authors:** Paul W Hill, Karina A Marsden, Davey L Jones

**Affiliations:** 1 School of Environment, Natural Resources and Geography, Bangor UniversityBangor, Gwynedd, LL57 2UW, UK

**Keywords:** dissolved organic nitrogen, endocytosis, mineralization, nitrogen cycle, oligopeptide, phagomixotrophy

## Abstract

**Summary:**

–The high degree to which plant roots compete with soil microbes for organic forms of nitrogen (N) is becoming increasingly apparent. This has culminated in the finding that plants may consume soil microbes as a source of N, but the functional significance of this process remains unknown.
–We used ^15^N- and ^14^C-labelled cultures of soil bacteria to measure rates of acquisition of microbes by sterile wheat roots and plants growing in soil. We compared these rates with acquisition of ^15^N delivered as nitrate, amino acid monomer (l-alanine) and short peptide (l-tetraalanine), and the rate of decomposition of [^14^C] microbes by indigenous soil microbiota.
–Acquisition of microbe ^15^N by both sterile roots and roots growing in soil was one to two orders of magnitude slower than acquisition of all other forms of ^15^N. Decomposition of microbes was fast enough to account for all ^15^N recovered, but approximately equal recovery of microbe ^14^C suggests that microbes entered roots intact.
–Uptake of soil microbes by wheat (*Triticum aestivum*) roots appears to take place in soil. If wheat is typical, the importance of this process to terrestrial N cycling is probably minor in comparison with fluxes of other forms of soil inorganic and organic N.

## Introduction

Nitrogen (N) is a key factor in the control of primary productivity in terrestrial systems (Vitousek & Howarth, 47; Liu & Greaver, 26). Consequently, a mechanistic understanding of the processes controlling its availability to photosynthetic organisms is crucial not only to provide a wider understanding of the N cycle, but also to make predictions of carbon (C) residence in the terrestrial biosphere. However, the paradigm of plant–soil N cycling has shifted repeatedly over the last few decades and significant breakthroughs continue to be made.

Historically, it was thought that plants derived all of their N nutrition from the inorganic forms of N, 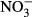 and 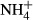. However, the principal form of N entering soils not receiving additions of inorganic fertilisers is protein derived from dead portions of plants, animals or microbes. Consequently, it was thought that protein must be cleaved by microbial extracellular proteases and taken up by microbes as amino acids before inorganic N could become available to plants. In many soils, soil microbes are C limited and often excrete much of the N acquired in amino acids as inorganic N (Schimel & Bennett, 44; Roberts *et al*., 41). By contrast, as photosynthetic organisms, plants generally have an abundant supply of C and are frequently limited by N. Thus, if plants acquire N only in inorganic forms, their competition with soil microbes for N-containing compounds is generally low. However, it is now widely accepted that plants are able to compete with soil microbes for more complex N-containing compounds, when proteins are cleaved only to amino acids (Borstlap, 2; Chapin *et al*., 4; Jones & Darrah, 20; Näsholm *et al*., 32; Paungfoo-Lonhienne *et al*., 36). Further, it has recently become apparent that plants and soil microbes are both able to acquire amino acids when proteins have been cleaved only to short peptides (Komarova *et al*., 24; Hill *et al*., 14,b,c, 15; Soper *et al*., 46; Farrell *et al*., 7). Due probably principally to methodological constraints and variation between ecosystems, the exact importance of organic N to plant N nutrition remains uncertain (Jones *et al*., 21; Näsholm *et al*., 32; Hill *et al*., 14; Paungfoo-Lonhienne *et al*., 36). Nevertheless, although the limiting element may differ between organisms, it is clear that fierce competition for soil organic N exists between plant roots and soil microbes.

In the quest for adequate N nutrition, some angiosperms have developed specialized structures to acquire nutrients via carnivory. Recent evidence has shown that entry of the prey-derived protein into the carnivorous plant can occur partially by endocytosis (Adlassnig *et al*., 1). More surprisingly, it has also been shown that intact proteins may be acquired endocytotically by nonspecialist, nonmycorrhizal plant roots (*Arabidopsis thaliana* and *Hakea actites*; Paungfoo-Lonhienne *et al*., 34).

Phagomixotrophic ingestion of intact microorganisms is widespread amongst algae and endocytosis of prokaryotes is the probable origin of photosynthesis in eukaryotes (Raven *et al*., 38). Although increasing numbers of plant endophytic microbes have been identified in a range of plant tissues including roots, until recently direct uptake and digestion of microbes by angiosperms for nutritional purposes has been unrecognized (Rosenblueth & Martinez-Romero, 42; Hardoim *et al*., 12; Reinhold-Hurek & Hurek, 39). However, it has recently been demonstrated that microbes are acquired by the roots of angiosperms and that their associated N can be used nutritionally by the plants (Paungfoo-Lonhienne *et al*., 35). This discovery represents a potential step change in our understanding of competition for resources between soil microbes and plants, and how N is cycled in the terrestrial biosphere. What is not currently clear is what the quantitative significance of this process is when compared with other routes of plant N acquisition from soil.

This investigation was predicated on the need to evaluate the significance of this new route of N acquisition to plants when growing alone in sterile media and in soil. As our model angiosperm we chose to use the widely investigated and agriculturally important crop plant, wheat (*Triticum aestivum*). Wheat has previously been shown to utilize N forms ranging in organism or protein decomposition state from short l-peptides to 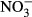 (Näsholm *et al*., 31; Hill *et al*., 18). We aimed to make a comparison of the rate of direct plant N acquisition from soil as intact bacteria with that as peptide, amino acid or inorganic N.

## Materials and Methods

### Soil

Agricultural Brown Earth soil (FAO classified as a Eutric Cambisol) was sampled (0–10 cm; *n *=* *4) from Henfaes Agricultural Research Station, Abergwyngregyn, Bangor (53°14′N, 4°01′W). Upon return to the laboratory the soil was sieved to pass 5 mm, removing stones, earthworms and vegetation. Soil solution was collected via centrifugal drainage (Giesler & Lundström, 11) and passed through a 1 kDa ultrafiltration membrane (Millipore). The collected soil solution was analysed for amino acids by the fluorometric method of Jones *et al*. (22), before and after hydrolysis in 6 M HCl at 105°C for 16 h under N_2_. Fresh soil was extracted with 0.5 M K_2_SO_4_ (1 : 5 w/v) and analysed for 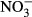 and 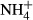, according to Miranda *et al*. (29) and Mulvaney (30), respectively, and total extractable C and N using a Shimadzu TOC-V-TN analyser (Shimadzu Corp., Kyoto, Japan). Microbial biomass C and N were determined by measuring total K_2_SO_4_-extractable C and N before and after chloroform fumigation according to Voroney *et al*. (48) (*K*_EC_ = 0.35; *K*_EN_ = 0.5). Soil moisture content was determined by oven drying at 105°C. The pH and electrical conductivity of the soil (1 : 5 w/v distilled water extract) were determined using standard electrodes. Soil total C and N were measured in a Carlo Erba NA 1500 Elemental Analyzer (Thermo Fisher Scientific, Milan, Italy).

### Culture of soil microbes

#### ^15^N-labelled microbes

Soil microbial cultures were obtained by adding *c*. 2 mg of soil to 50 ml of sterile (0.2 μm filtered) nutrient solution, in a sterile 250 ml Erlenmeyer flask, with a foam stopper. The nutrient solution contained cycloheximide (10 μg ml^−1^) to minimize growth of hyphal fungi, and the following macronutrients (in mM): KH_2_PO_4_, 8.0; MgSO_4_, 2.0; CaCl_2_, 0.2; KNO_3_, 1.0; glycine, 2.0. Glucose (50 mM) was added as a C source and micronutrients were included as Kao and Michayluk vitamin mixture (Sigma-Aldrich, Gillingham, UK) at a concentration of 1 : 100 (v/v). The soil and nutrient solution were incubated aerobically on a rotary shaker at 75 rpm at 20°C. After 3 d, 1 ml of the microbial suspension was added to 50 ml of new nutrient solution, as above, except without cycloheximide. After a further 3 d of incubation, 1 ml of the microbial suspension was sub-sampled and suspended in 50 ml of new media containing K^15^NO_3_, in replacement of the unlabelled KNO_3_, at the same concentration as above. This last step was repeated to ensure that microbial N was effectively all ^15^N. After the final incubation, the microbial suspension was centrifuged at 4500 ***g*** for 10 min. The supernatant was discarded and the pellet was washed with 0.01 M CaCl_2_. The washing procedure was repeated twice, followed by re-suspension of the pellet in 20 ml of 0.01 M CaCl_2_. Total organic C and N of the microbial suspension were measured on the Shimadzu TOC-V-TN analyser. The suspension was subsequently diluted in 0.01 M CaCl_2_ to make a final concentration of 1 mmol N l^−1^ (*c*. 6 mmol C l^−1^). The solution was used immediately following preparation. Haemocytometer counts found microbe numbers in the injected solution to be *c*. 8 × 10^6^ cells ml^−1^.

#### ^14^C-labelled microbes

In order to sensitively measure plant incorporation of microbial C, a second culture of ^14^C-labelled microbes was prepared. Due to constraints on stable isotope analysis, it was not possible to dual-label microbes with ^15^N and ^14^C. Initially the ^14^C-labelled microbes were prepared in the same way as the ^15^N-labelled microbes. However, after the second incubation 2 MBq of [U^−14^C] glucose was added to the nutrient solution. To ensure high ^14^C incorporation, the unlabelled glucose in the nutrient solution was reduced from 50 mM to 50 μM. After 2 d of incubation the washing procedure described above was carried out. The solution was diluted to 1 mmol N l^−1^, and the ^14^C activity in the microbial suspension was determined by liquid scintillation counting in a Wallac 1404 scintillation counter (Perkin-Elmer Life Sciences, Boston, MA, USA) after mixing with Scintisafe scintillation cocktail (Fisher Scientific, Loughborough, UK).

### Uptake of N forms by plants growing in soil

Seeds of wheat (*Triticum aestivum* L. var. AC Barrie) were sown singly into rhizotubes (240 mm long; internal diameter 8 mm; Owen & Jones, 33) containing 12 g of field-moist soil (Table 1). The plants were grown at 15°C, 70% relative humidity and 16 h photoperiod (*c*. 500 μmol photons m^−2^ s^−1^ PAR) until the third leaf stage (root weight 0.03 ± 0.002 g DW; mean ± SEM; *n *=* *23). Injections of 0.5 ml of the N-treatments were made into the rhizosphere halfway up the rhizotube, (i.e. at a depth of 12 cm) using sterile 1 ml polypropylene syringes with 18G needles. Rhizotubes allowed injection of solutes and suspended microbes directly into the rhizosphere, thereby reducing possible confounding effects of differing mobility of N forms in the soil. Solutions injected were 1 mM K^15^NO_3_, 1 mM [^13^C^15^N] l-alanine (C_3_H_7_NO_2_), 250 μM [^13^C^15^N] l-tetraalanine (C_12_H_22_N_4_O_5_) and ^14^C or ^15^N labelled microbes (1 mmol N l^−1^). Four plants, selected randomly, were supplied with each of the N treatments. One hour after ^15^N injection, plants were removed from rhizotubes and roots carefully washed with *c*. 1 l distilled water and *c*. 200 ml 0.01 M CaCl_2_. After drying (80°C) root and shoot were weighed. ^15^N-labelled material was ground and analysed in a Thermo Finnigan Delta Plus XL continuous flow mass spectrometer (Thermo Fisher Scientific, Waltham, MA, USA). ^14^C-labelled material was combusted in an OX400 Biological Oxidiser (Harvey Instruments Corp., Hillsdale, NJ, USA). Liberated ^14^CO_2_ was captured in Oxosol Scintillant (National Diagnostics, Atlanta, GA, USA) and ^14^C activity measured by liquid scintillation counting.

**Table:** Basic properties of soil used to fill rhizotubes before growth of plants or culture of microbes

Soil property	
Moisture content (g g^−1^ DW)	0.28 ± 0.04
pH	6.5 ± 0.04
Electrical conductivity (μS cm^−1^)	22 ± 1.6
Total C (mg g^−1^ DW)	34 ± 3*
Total N (mg g^−1^ DW)	0.54 ± 0.08*
Total soluble C (μmol g^−1^ DW)	10.3 ± 0.9
Total soluble N (μmol g^−1^ DW)	3.3 ± 0.3
Microbial C (μmol g^−1^ DW)	267 ± 42
Microbial N (μmol g^−1^ DW)	39 ± 6
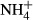 (nmol g^−1^ DW)	83 ± 12
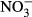 (nmol g^−1^ DW)	249 ± 7
Soil solution amino acids (μmol N l^−1^)	42 ± 9
Soil solution < 1 kDa peptides (μmol N l^−1^)	107 ± 34

1 Values are mean ± SEM; *n *=* *4.

2 Determined for this soil by Farrell *et al*. (6).

Three plants were retained as ^15^N and ^13^C background enrichment controls. Three rhizotubes were injected with 0.5 ml of blue ink to estimate how much root and soil came into contact with the injected solution. The resulting blue section was cut out of the rhizotube and roots were washed, dried (80°C) and weighed. A further four rhizotubes had the same section cut out to determine pre-injection pools of soil soluble N with which injected solutions mixed. Soil from these sections was extracted for 5 min in 6 ml of de-ionized water. Nitrate, amino acids and short peptides (< 1 kDa) in the extract were measured as for the starting soil or soil solution. Roots from rhizotubes used to determine soil N pools were washed, cleared in 10% w/v KOH, stained in 0.25% w/v aniline blue and inspected for mycorrhizal colonization by light microscopy after destaining and mounting in 80% w/v lactic acid.

### Uptake of N forms by sterile plants

Seeds were surface sterilized in sodium hypochlorite (10–14% available chlorine) with one drop of Tween-20, for 10 min followed by 80% v/v ethanol for 1 min. Seeds were then washed thoroughly in sterile distilled water. Seeds were placed on agar plates to germinate, following which, they were transferred to 114 × 86 × 102 mm Phytatrays (Sigma-Aldrich). Phytatrays contained 10 g of sterile (autoclaved) perlite, with 60 ml of sterile 50% Long Ashton nutrient solution (containing Na-metasilicate). Plants were kept under the same conditions as the wheat grown in soil. Upon reaching the third leaf stage, the plants were removed from the perlite and rinsed thoroughly with distilled water and 0.01 M CaCl_2_ as for plants grown in soil. The intact roots of three plants per treatment were submerged individually in sterile containers containing 4 ml of either 1 mM K^15^NO_3_, 1 mM [^13^C^15^N] l-alanine, 250 μM [^13^C^15^N] l-tetraalanine or ^15^N labelled microbes (1 mmol N l^−1^). Solutions not containing cultured microbes were sterilized by filtration to 0.2 μm before use. After 1 h under the same conditions as when growing, plants were removed from solutions and washed, dried and weighed as above. Root and shoot were ground together before mass spectrometer analysis.

The diameter of fresh roots (*n *=* *10) of three plants grown in sterile culture was measured using a micrometer. Root length was measured with a ruler. These roots were subsequently dried (80°C) and used to determine the approximate relationship between root length and dry weight.

### Consumption of added microbes by the indigenous soil microbial community

In order to evaluate the rate at which microbes added to soil were predated and decomposed by pre-existing soil microbes, ^14^C-labelled microbes were added to soil and their decomposition to ^14^CO_2_ measured according to Hill *et al*. (13). Briefly, 2 g of soil was placed in each of three 10 ml glass tubes and 83 μl of the washed ^14^C-labelled microbe suspension were added to the surface (matching the overall ratio of solution to soil in rhizotube injections). Air was drawn over the soil at a rate of *c*. 100 ml min^−1^ and ^14^CO_2_ was captured in two 3 ml vials of 0.1 M NaOH connected in series (described in detail in Hill *et al*., 16). Vials of NaOH were changed 1, 5, 10, 20, 40 and 60 min after addition of the microbes to soil. There was no potential for significant loss of ^14^CO_2_ during changing of NaOH as pumping was suspended for this period. Captured ^14^C was measured by liquid scintillation counting after mixing NaOH solution with Scintisafe scintillation cocktail. To account for ^14^CO_2_ lost in respiration from the microbial culture alone, the experiment was repeated with 1 ml of culture solution in sterile tubes without soil.

### Statistical analysis

Data were analysed by One-way ANOVA with LSD *post-hoc* test (SPSS v14; SPSS, Chicago, IL, USA) after testing for normality and homogeneity of variance with Shapiro-Wilk and Levene's test, respectively. Data not normally distributed or without homogeneity of variance were log_10_-transformed before analysis. Following transformation all data were normally distributed and had homogeneity of variance. Statistical differences were accepted at *P *<* *0.05. All presented probabilities are for *post hoc* comparisons.

## Results

### Plants grown in sterile culture

**Figure 1:**
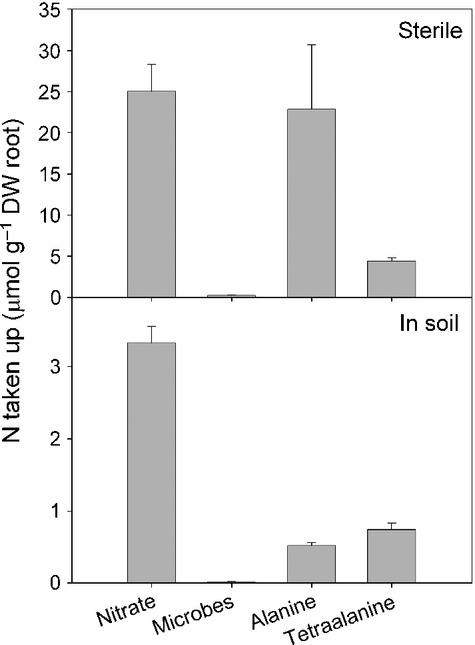
Rate of uptake of ^15^N supplied as nitrate, l-alanine, l-tetraalanine or ^15^N-labelled microbial culture to sterile wheat (*Triticum aestivum*) plant roots and roots of plants growing in soil. Values are mean ± SEM; *n *=* *3 for sterile plants and *n *=* *4 for plants grown in soil. Values for both plants in sterile culture and plants grown in soil assume uptake over the entire root system.

**Figure 2:**
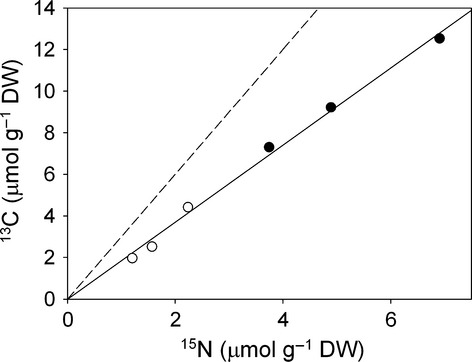
Concentrations of ^15^N and ^13^C recovered in tissues of wheat (*Triticum aestivum*) plants grown in sterile culture after supply of ^15^N^13^C-dual-labelled l-alanine or l-tetraalanine. Values are data for individual plants. The solid line is the line of best fit using data for plants supplied with both alanine and tetraalanine (*r*^2^* *=* *0.994; slope = 1.85). The dashed line represents the relationship between ^15^N and ^13^C in the compounds supplied to roots. Closed circles, alanine; open circles, tetraalanine.

Uptake of ^15^N by sterile plant roots when in nonmicrobial forms was 18-, to *c*. 100-fold faster (tetraalanine, and alanine and nitrate, respectively; *P *<* *0.001; Fig. 1) than when present in a microbial form. Uptake of ^15^N-nitrate took place at the same rate as ^15^N-alanine and ^15^N uptake was approximately five-fold faster (*P *≤* *0.003) in both these forms than as tetraalanine. Recovered ^15^N accounted for 5.1 ± 0.6*%* of nitrate, 3.1 ± 0.2% of alanine, 0.84 ± 0.1% of tetraalanine and 0.046 ± 0.02% of microbe ^15^N supplied in the 4 ml of solution. The ratio of the proportion of ^13^C recovered to the proportion of ^15^N recovered from organic forms of N was almost identical in plants supplied with alanine and those supplied with tetraalanine (0.63 ± 0.01 and 0.58 ± 0.04, respectively; mean ± SEM; *n *=* *3; Fig. 2).

Roots within the microcosms were predominantly second-order laterals and average root diameter was 0.3 ± 0.03 mm (mean ± SEM; *n *=* *10). The specific root length was 63 ± 8 m g^−1^ DW.

### Plants grown in soil

As in sterile culture, root uptake of ^15^N added as nitrate, alanine or tetraalanine in plants grown in soil was much greater (*P *<* *0.001; 208-, 32- and 46-fold, respectively; Fig. 1) than when added as the microbial suspension. Uptake of ^15^N by roots of plants supplied with nitrate was six- and five-fold higher (*P *<* *0.001) than in plants supplied with alanine or tetraalanine, respectively, which were not different from each other. After 1 h 15.8 ± 2% of the added nitrate ^15^N, 4.5 *±* 0.6% of the alanine ^15^N and 3.9 ± 0.5% of the tetraalanine ^15^N was recovered in plants, but only 0.079 ± 0.008% of the microbial ^15^N. Post-uptake partitioning of ^15^N also differed (*P *<* *0.02) between plants supplied with the different forms of N. The ratio of root ^15^N to shoot ^15^N was 1.2 ± 0.1 for plants supplied with microbial N, 2.4 ± 0.6 for those supplied with nitrate, 9.8 ± 1.5 for those supplied with tetraalanine and 18 ± 2.7 for plants supplied with alanine. Ratios for plants receiving nitrate and those receiving microbes were not statistically different.

Data for ^13^C were much more variable than ^15^N data in plants receiving dual-labelled compounds. This was especially true of shoots where ^13^C recovery was only 26 ± 10 or 15 ± 7% (mean ± SEM; *n *=* *3; alanine and tetraalanine, respectively) of that recovered in roots. Recovery of ^13^C and ^15^N from dual-labelled compounds in plants grown in soil receiving N as both alanine and tetraalanine were not linearly correlated. The ratio of the proportion of ^13^C recovered to the proportion of ^15^N recovered was 0.6 in plants receiving alanine and 0.4 in those receiving tetraalanine, but we suggest that values for the ratio of ^13^C to ^15^N for plants grown in soil should be interpreted with caution. Recovered microbial ^15^N and ^14^C could not be correlated as they were separate samples. The mean value for recovery of ^14^C was, however, similar to that for ^15^N at 0.070 ± 0.02% of that injected.

From injections of ink into rhizotubes it was estimated that added solutes mixed with the soil of a 7 cm section of the rhizotube. These 7 cm sections contained 11.6 ± 0.8 mg root DW (approximately one-third of the total root in the rhizotube), 15.7 ± 9 nmol 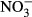, 47.4 ± 8 nmol free amino acid N and 255 ± 34 nmol short (< 1 kDa) peptide N (mean ± SEM; *n *=* *3 for root DW and *n *=* *4 for solute concentrations). If it is assumed that the root biomass in contact with labelled solutes was a constant 11.6 mg DW, pre-existing soil solutes mixed completely with labelled solutes, and l-alanine and l-tetraalanine are typical of all soil solution amino acids and short peptides, rates of uptake for alanine and tetraalanine remain statistically the same at 2.1 ± 0.3 and 2.5 ± 0.3 μmol N g^−1^ DW root h^−1^, respectively. Uptake of nitrate is 7.0 ± 0.8 μmol N g^−1^ DW root h^−1^, making it approximately three-fold greater (*P *<* *0.001) than amino acid and peptide N uptake.

No evidence of mycorrhizal infection could be found on roots.

Over the hour of incubation, 3.1 ± 0.2% of the added microbial ^14^C was mineralized to ^14^CO_2_ after accounting for that lost in respiration from the added microbes themselves (0.3 ± 0.06%).

## Discussion

### Acquisition of dissolved forms of N

In sterile hydroponic culture, nitrate ^15^N was taken up at the same rate as alanine ^15^N and five times faster than tetraalanine ^15^N. In contrast to the equal rate of nitrate and amino acid acquisition in sterile culture, competition from microbes caused a large reduction in acquisition of alanine ^15^N relative to that of nitrate ^15^N when the plants were grown in nonsterile soil. Previous investigations have suggested some form of preference of soil microbes for l-peptides over the amino acid monomer (Farrell *et al*., 8, 7; Hill *et al*., 17, 15). Consequently, it was surprising that while ^15^N uptake of alanine was considerably reduced in soil, the rate of acquisition of ^15^N as tetraalanine relative to nitrate by roots in soil was similar to that in sterile solution. Further, what evidence there is suggests that soil solution concentrations of amino acids bound in short peptides are generally higher than those of free amino acids (Farrell *et al*., 6; Hill *et al*., 14,b,c; Table 1). This may indicate that acquisition of N as short l-peptides by wheat growing in agricultural soil exceeds that as l-amino acid monomers.

Realistic evaluation of the importance of different forms of soil N to plant nutrition presents formidable experimental problems, especially for organic forms. Even when attempting to determine relative fluxes, it is necessary to consider a range of potential caveats in the interpretation of data. We found no statistically significant correlation between recovered ^13^C and ^15^N for plants grown in soil. This may principally be a consequence of variation caused by the dilution of the ^13^C taken up as intact alanine or tetraalanine in a *c*. 15 000-fold larger pool of plant carbon with a variable ^13^C content. Some support for this view is provided by the much larger recovery of ^13^C in roots than in shoots and the fact that rates of recovery of ^13^C in plants grown in soil did not depart dramatically from those which would be expected from recovery of ^15^N after losses in respiration (Hill *et al*., 18). Nevertheless, we cannot exclude the possibility that some or even all of the amino acid and/or peptide ^15^N was acquired by plants following prior mineralization by soil microbes. Taking account of isotopic pool dilution in pre-existing pools of soil N also relies on various assumptions. Even if it is clear what proportion of pre-existing soil soluble N is mixed with, differing flux rates through soil pools for the different forms of N add uncertainty. For instance, the residence time of l-amino acids and short l-peptides in soil is probably only of the order of a few minutes (Hill *et al*., 15). Thus, if acquired intact, the flux of N into plants as amino acid or peptide relative to that of forms of N which are less desirable to most soil microbes, such as nitrate, is probably underestimated with a chase period of an hour (Jones *et al*., 21). If amino acid and peptide N are only acquired following mineralization, recovery of ^15^N in plants would be likely to increase with the chase period. In this case, the actual flux of N derived from mineralization of organic forms would be underestimated due to dilution by pre-existing inorganic N and this dilution would probably increase with residence time. Many uncertainties relating to solute production sites and mobility in soil may also be of considerable importance in the design of experiments and interpretation of experimental data. Further, we know little about the composition of individual peptides in soil solution. For instance, 160 000 possible tetrapeptides may be formed from 20 common protein amino acids. To date, the availability to plants of very few peptides has been investigated. Consequently, after correction for pool dilution fluxes of, particularly peptide, N from soil to plant must be interpreted with some caution.

### Acquisition of N as microbes

Nitrogen delivered to plant roots as a microbial culture was acquired both by plants with sterile roots and those growing in soil. In both cases the nitrate, amino acid monomer and tetrapeptide forms of N were taken up more than an order of magnitude faster than the microbial suspension. If all of the measured microbial ^15^N acquired by plants was acquired as intact microbes, our results suggest that sterile roots of plants ingested *c*. 2 × 10^6^ microbial cells g^−1^ root DW h^−1^. This suggests that microbes were ingested at a rate of *c*. 32 000 cells m^−1^ root length h^−1^ or 35 cells mm^−2^ root surface area h^−1^, although this does not take account of fine root structure such as root hairs, which may have a role in microbe acquisition (Paungfoo-Lonhienne *et al*., 35; Mercado-Blanco & Prieto, 27). To match the rate of N uptake as nitrate or alanine from sterile solution, plants would need to ingest *c*. 2 × 10^8^ cells g^−1^ root DW h^−1^ and *c*. 4 × 10^7^ to match N uptake as tetraalanine. When growing in soil, ^15^N recovery suggests that plants ingested *c*. 390 000 cells g^−1^ root DW h^−1^; *c*. 6 cells mm^−2^ root surface area h^−1^ (assuming a constant 11.6 mg of root in a 7 cm section of rhizotube was accessed by injected microbes). If previous estimates of numbers of bacteria on wheat roots are typical and it is assumed that labelled microbes mixed homogeneously with existing rhizoplane microbes, this suggests that rates of uptake were *c*. 975 000 cells g^−1^ root DW h^−1^: *c*. 15 cells mm^−2^ root surface area h^−1^; *c*. 4.5% of the standing rhizoplane bacterial biomass d^−1^ (Liljeroth, 25). If it is assumed that injected cells mixed with all bacteria on the rhizoplane and in the rhizo-sphere, this value rises to *c*. 10^6^ cells g^−1^ root DW h^−1^ or *c*. 16 cells mm^−2^ root surface area h^−1^. Nevertheless, this flux of N into roots still represents only a maximum of *c*. 6% of the flux of other forms of N when they are similarly corrected for pool dilution. There are uncertainties in the dilution of amino acid, peptide and nitrate in pre-existing soil pools. However, very poor understanding of the process of direct microbe uptake by roots means that the size of the pool with which added microbes mixed is very difficult to establish. Consequently, our estimates of uptake of N as microbes are probably subject to the greatest uncertainty.

Three percent of the ^14^C added to soil as microbes was mineralized to ^14^CO_2_ within an hour. This was around a fifth of the ^14^C likely to be mineralized to ^14^CO_2_ if added to soil of this type as glucose (Hill *et al*., 13). Nevertheless, respired ^14^CO_2_ was an almost 40-fold greater proportion of the microbial biomass ^14^C than the proportion of the microbial ^15^N which was recovered in plants (0.08%). Similarly, the 0.3% of the microbial ^14^C respired by the living microbes alone was a six times greater proportion of microbial ^14^C than the 0.05% of microbial ^15^N recovered in sterile plants. This may indicate that in both soil and sterile solutions, the microbial ^15^N recovered in plants was taken up as inorganic ^15^N after microbial mineralization. However, although there is some uncertainty inherent in the measurement of ^14^C and ^15^N in separate plants, the close agreement between values for recovery of microbial ^15^N and ^14^C in plants growing in soil strongly suggests that ^15^N was not acquired only in inorganic forms. We cannot completely exclude the possibility that both N and C were taken up as organic forms of N following prior lysis of microbes. In our opinion this seems unlikely to account for the entire flux of ^14^C and ^15^N, as that would necessitate the maintenance of the overall microbial ^14^C to ^15^N ratio in the organic forms of N taken up after any losses of C in respiration. Nevertheless, post-lysis, or even post-mineralization, plant uptake probably accounts for part of the flux of microbial N into roots.

Many living endophytes exist in plants, although our knowledge of how widespread the ability to survive within plants is amongst soil microbes is largely restricted to studies on a few species (Hardoim *et al*., 12; Ryan *et al*., 43; Reinhold-Hurek & Hurek, 39). Our measurements of incorporation of microbial ^15^N and ^14^C cannot distinguish between microbes internalized and metabolized within root cells and those continuing to survive within the plant, that is within the apoplast or root wounds (Gantar, 10; Hardoim *et al*., 12; Paungfoo-Lonhienne *et al*., 35). The close agreement between the ^15^N and ^14^C recovery from the microbial cells contrasts with the at least 40% of amino acid and peptide ^13^C which was rapidly metabolized and lost in respiration. This may also indicate that microbes were not metabolized by the plant after uptake. Further, in some cases, endophytes move from root to shoot within the plant without apparent attack or digestion by the host with bacterial movement from the root epidermis to the stele occurring via the apoplast (Reinhold-Hurek *et al*., 40; Rosenblueth & Martinez-Romero, 42; Deering *et al*., 5). Consequently, ^15^N recovery in the shoot cannot be unequivocally attributed to degradation of microbes in the root, with subsequent transport of ^15^N to the shoot (Paungfoo-Lonhienne *et al*., 35). However, even if plant degradation of microbes took place more slowly than other forms of organic N and too slowly to be very obvious over the hour of experiment duration, eventual death and decomposition of some microbes within the plant does seem likely.

Assuming that bacteria can be internalized within root cells, it raises questions about the mechanism of cytophagocytosis and to what extent this is under direct control of the plant (Hardoim *et al*., 12; Paungfoo-Lonhienne *et al*., 35). Due to the small pore size of the cell wall (< 10 nm) relative to the size of bacterial cells (*c*. 1000 nm), internalization can only occur by loosening/digestion of the cell wall in mature root regions or possibly at weak points in actively growing cells (Reinhold-Hurek *et al*., 40; Miralles *et al*., 28). If the plant is actively undertaking this process to acquire N, strong selection for nonharmful bacteria is expected, bypassing myriad host-defence processes (Kogel *et al*., 23; Rosenblueth & Martinez-Romero, 42; Hückelhoven, 19). Perhaps more probable is that many bacterial cells are taken up passively as has been demonstrated for a range of inert micro- and nano-particulates (Solomon & Matthews, 45; Miralles *et al*., 28). However, whilst it is clear from many studies that live bacteria can rapidly enter and survive in the endorhizosphere, little evidence exists for the passive uptake of dead cells into the apoplast (Quadt-Hallmann *et al*., 37; Hardoim *et al*., 12).

Although considerable uncertainty surrounding mechanisms remains, our results suggest that acquisition of microbes from soil by wheat roots, with subsequent translocation of N, does take place. Thus, if this is actively undertaken, all three plant species investigated to date, *Arabidopsis thaliana*, *Solanum lycopersicum* and *Triticum aestivum*, appear to have this capacity (Paungfoo-Lonhienne *et al*., 35). However, if wheat proves to be typical, low rates of uptake of N as intact microbial cells in comparison with uptake of common inorganic and organic forms of soil N suggest that the importance of this process to overall plant N nutrition is minor. Use of other forms of organic N is often considered to be most important in environments where N mineralization is slow (Chapin *et al*., 4; Schimel & Bennett, 44; Näsholm *et al*., 32; Hill *et al*., 14). Similarly, wider investigation may establish that uptake and digestion of soil microbes has high functional importance in some ecosystems; perhaps when free-living diazotrophs are abundant in an N-limited rhizosphere. In these respects, the use of microbes may be more significant in some angiosperms than it is in a highly-bred agricultural plant such as wheat. Of course, it may also be that microbes are primarily consumed as a source of some nutrient other than N, or that their consumption is only of transient importance.

Although each form of N differs in its mobility in soil, strong sorption of microbial cells to soil particles results in a slow rate of diffusion to the root surface (Foppen *et al*., 9). Consequently, most microbes available to plants live and reproduce very close to roots and derive much of their nutrition from roots, for example, as exudates (Liljeroth, 25; Brimecombe *et al*., 3). This may mean that active consumption of these microbes primarily represents a mechanism by which plants recover lost nutrients, as has been proposed for some organic solutes (Jones *et al*., 21).

### Conclusion

Uncertainty still surrounds the extent to which plants can acquire different forms of organic N from soil. Previous studies undertaken in sterile hydroponic culture have clearly demonstrated the potential for plant roots to take up intact microbial cells, but the functional significance of this process in soil environments remains unknown. The results presented here for wheat plants grown in both hydroponics and in soil strongly suggest that the rate of uptake of N as intact microbial cells is very low in comparison with uptake of common inorganic and organic forms of soil N. Although wider investigation is needed and other functions cannot be excluded, this relatively low incorporation of microbial N suggests that digestion of soil microbes probably represents only a small component of overall plant N acquisition.
